# Development of an Electronic Health Record Self-Referral Tool for Lung Cancer Screening: One-Group Posttest Study

**DOI:** 10.2196/53159

**Published:** 2024-06-12

**Authors:** Garrett S Stang, Nichole T Tanner, Ashley Hatch, Jakarri Godbolt, Benjamin A Toll, Alana M Rojewski

**Affiliations:** 1 Department of Behavioral and Social Sciences Brown University School of Public Health Providence, RI United States; 2 Hollings Cancer Center Medical University of South Carolina Charleston, SC United States

**Keywords:** lung cancer screening, LCS, electronic health records, EHR, Health Belief Model, HBM, self-refer, tobacco treatment, cancer screening, development, self-referral tool, electronic health record, decision-making

## Abstract

**Background:**

Approximately 14 million individuals in the United States are eligible for lung cancer screening (LCS), but only 5.8% completed screening in 2021. Given the low uptake despite the potential great health benefit of LCS, interventions aimed at increasing uptake are warranted. The use of a patient-facing electronic health record (EHR) patient portal direct messaging tool offers a new opportunity to both engage eligible patients in preventative screening and provide a unique referral pathway for tobacco treatment.

**Objective:**

This study sought to develop and pilot an EHR patient-facing self-referral tool for an established LCS program in an academic medical center.

**Methods:**

Guided by constructs of the Health Belief Model associated with LCS uptake (eg, knowledge and self-efficacy), formative development of an EHR-delivered engagement message, infographic, and self-referring survey was conducted. The survey submits eligible self-reported patient information to a scheduler for the LCS program. The materials were pretested using an interviewer-administered mixed methods survey captured through venue-day-time sampling in 5 network-affiliated pulmonology clinics. Materials were then integrated into the secure patient messaging feature in the EHR system. Next, a one-group posttest quality improvement pilot test was conducted.

**Results:**

A total of 17 individuals presenting for lung screening shared-decision visits completed the pretest survey. More than half were newly referred for LCS (n=10, 60%), and the remaining were returning patients. When asked if they would use a self-referring tool through their EHR messaging portal, 94% (n=16) reported yes. In it, 15 participants provided oral feedback that led to refinement in the tool and infographic prior to pilot-testing. When the initial application of the tool was sent to a convenience sample of 150 random patients, 13% (n=20) opened the self-referring survey. Of the 20 who completed the pilot survey, 45% (n=9) were eligible for LCS based on self-reported smoking data. A total of 3 self-referring individuals scheduled an LCS.

**Conclusions:**

Pretest and initial application data suggest this tool is a positive stimulus to trigger the decision-making process to engage in a self-referral process to LCS among eligible patients. This self-referral tool may increase the number of patients engaging in LCS and could also be used to aid in self-referral to other preventative health screenings. This tool has implications for clinical practice. Tobacco treatment clinical services or health care systems should consider using EHR messaging for LCS self-referral. This approach may be cost-effective to improve LCS engagement and uptake. Additional referral pathways could be built into this EHR tool to not only refer patients who currently smoke to LCS but also simultaneously trigger a referral to clinical tobacco treatment.

## Introduction

Lung cancer is the second most common cancer worldwide and the leading cause of cancer-related death in the United States, with higher mortality rates than breast, colon, and prostate cancer combined [1-3]. High-quality evidence demonstrates a 20%-35% reduction in lung cancer mortality by screening those at high risk of lung cancer based on age and smoking history [4,5]. Based on this, the US Preventative Services Task Force has given a grade B recommendation in favor of screening with low-dose computed tomography for individuals aged 50 to 80 years who currently smoke or have quit within the past 15 years with a minimum 20-pack-year smoking following a shared decision-making visit [1,6]. Despite the benefits of screening and an estimated number of 14 million individuals in the United States who are eligible for lung cancer screening (LCS) [7], uptake of LCS has been low with only 12.8% having undergone screening [8]. Identifying approaches to increase LCS uptake would greatly benefit public health [9].

Barriers to LCS uptake have been identified at the system, provider, and patient levels. At the provider level, lack of familiarity with eligibility criteria, insufficient time or knowledge to conduct shared decision-making, skepticism about the benefits of screening, and familiarity with managing screen-detected findings have all been identified as barriers to uptake [10]. Patient-level barriers to LCS engagement include a need for more awareness of screening eligibility or programs, cost concerns, insurance status, and challenges in accessing imaging sites [10,11]. In addition, LCS is the first preventative screening to target poor health behavior (ie, smoking), and this population is less likely to have a primary care provider or engage in other screening services [11]. Thus, reducing barriers to LCS among this high-risk population who may not be engaging in health-promoting behaviors is needed to increase uptake.

This study aimed to develop an intervention to target the individual behavioral factors associated with low LCS uptake. A significant contributor to poor uptake is poor knowledge of the benefits of screening (eg, perceived benefit) and a lack of perceived susceptibility and severity of lung cancer among screening-eligible patients [12-16]. Guided by the Health Belief Model (HBM) and leveraging an electronic health record (EHR) patient portal direct messaging feature, the tool is a multilevel theory-based conceptual model that targets behavior change in LCS uptake to engage potentially eligible patients in preventative screening [17,18].

## Methods

### Theory-Guided Development

The HBM guided the development of the tool components. Research has shown that constructs within the HBM are associated with LCS uptake [14]. The tool components were designed to target knowledge of screening eligibility, the perceived threat of lung cancer, perceived benefits of LCS, self-efficacy, and prompt a cue to action for LCS self-referral.

### Self-Referral Tool Design

#### Overview

The tool was designed to be integrated into the EHR (Epic Systems); Epic Systems is a third-party, cloud-based EHR [19]. We used the patient-facing secure messaging application (MyChart) for self-referral to the medical center’s centralized LCS program. A centralized lung screening program uses a dedicated lung screening navigator and a team that verifies eligibility, conducts shared decision-making, initiates tobacco treatment, orders and schedules imaging, manages results, and ensures adherence to follow-up. The tool components include an initial engagement message, a questionnaire, and an infographic highlighting the danger of lung cancer and LCS eligibility.

#### Initial Engagement Message

**Figure 1:**
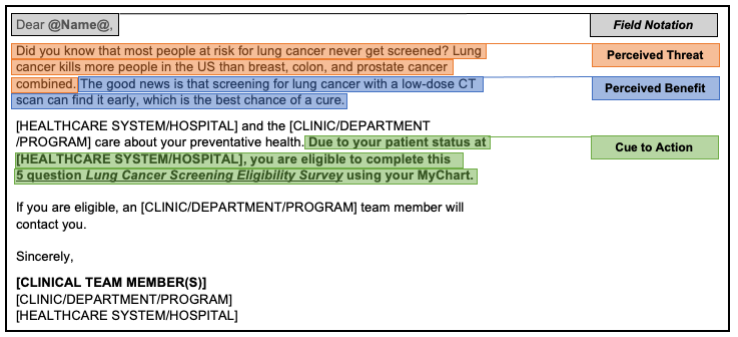
Initial MyChart engagement message mapped to HBM constructs. CT: computed tomography; HBM: Health Belief Model.

The initial engagement message conveyed the threat of lung cancer, the benefit of being screened, and a cue to action to self-assess their LCS eligibility. The initial engagement message mapped to the HBM constructs is presented in Figure 1.

#### Questionnaire

**Figure 2:**
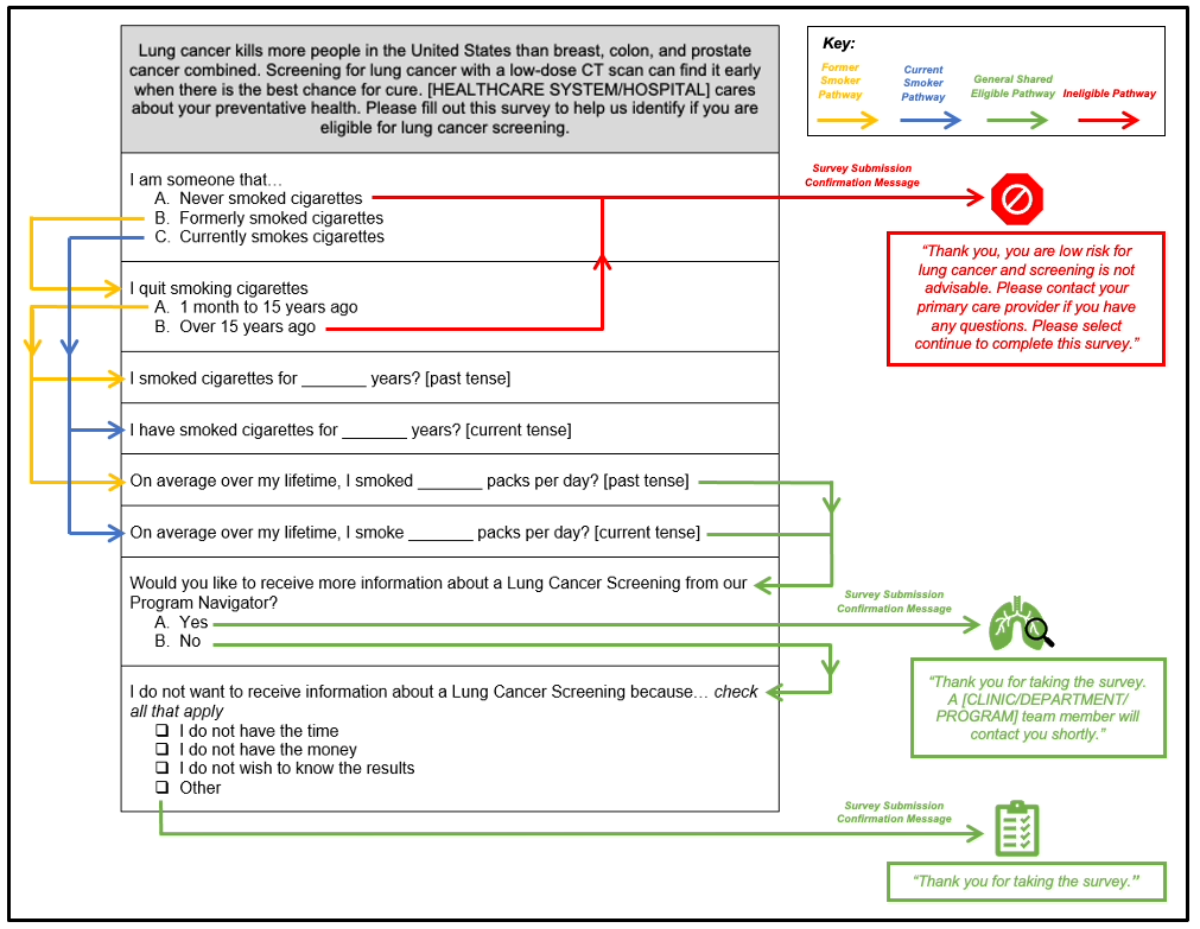
MyChart lung cancer screening questionnaire branching logic. CT: computed tomography.

The MyChart-delivered questionnaire presents patients with the opportunity to assess their eligibility for LCS and self-refer to the LCS scheduler. The questionnaire assesses the individual’s self-reported smoking status, years smoked, and packs per day, which map to the 3 LCS eligibility criteria. Questions and responses were written at a presumed accepted general health literacy level. Figure 2 displays the questionnaire and associated branching logic. The results of this survey would then be sent to the LCS scheduler directly as an acute care LCS referral. Once the referral is received, the LCS scheduler will confirm eligibility and contact the patient directly to schedule their LCS.

#### Infographic

An infographic was created to expand on the emphasized perceived threat of lung cancer established in the initial engagement message. We determined that including an infographic in the tool lineup would increase questionnaire completion efforts by allowing individuals to visualize relevant statistics in an easily digestible format. The infographic mapped to the HBM constructs is presented in Multimedia Appendix 1.

### Self-Referral Tool Pretest

#### Overview

The tool components were evaluated using a venue-day-time sampling interviewer-administered survey to gather feedback for iterative tool refinement prior to implementation.

#### Study Setting and Participants

Tool evaluation was conducted by participants meeting eligibility for LCS being seen in the clinic as a new referral for LCS or returning patient with an active Epic MyChart account. Over 4 weeks, predetermined days and times were selected for data collection in 5 separate pulmonology clinics in the tri-county region of South Carolina where this investigation was conducted (Berkley, Charleston, and Dorchester).

#### Study Procedures

Eligible patients were identified through manual EHR review. Following the completion of the scheduled appointment, the LCS provider invited the patient to participate in the study. Those who agreed to participate then met with and gave verbal informed consent to the clinical research assistant (CRA). The participant was presented with copies of the self-referral questionnaire and the infographic. Then, a semistructured interview and survey were conducted to gauge their response to the tool components.

#### Data Collection and Analysis

The survey was a mixed methods instrument that collected quantitative and qualitative data through open-ended questions. The survey measured health literacy, usability, perceived susceptibility, and self-efficacy using a 5-point Likert scale (1: strongly agree, 2: agree, 3: neutral, 4: disagree, and 5: strongly disagree). The survey also evaluated their overall willingness to complete the self-referral survey (yes, no, and unsure) and the likelihood of completing the self-referral questionnaire without the accompanying message and infographic (yes—I would complete any questionnaire sent to me via MyChart, yes—but I would prefer to get those too, no—but I would if I also got the letter and infographic, no—I would not complete the questionnaire in MyChart, and unsure). The qualitative free-responses items allowed participants to provide additional feedback on each tool component. Nonidentifiable demographic data were also collected from a manual EHR chart review. The CRA took field notes of observations and quotes. Standard descriptive statistics were conducted to summarize quantitative variables. These analyses included a mean and SD for age and percentages for the remaining categorical variables. Qualitative components (field notes and free responses) were analyzed using a content, thematic approach via inductive coding.

### EHR Integration

Following pretest analysis, the self-referral tool components were refined and integrated into the Epic system for broader testing. The self-referral tool was integrated into the Epic system secure patient messaging feature (ie, In Basket) as a general questionnaire task item. The initial engagement message was formatted as a SmartText option; this created a fixed message. The only modifiable element in the message was the patient’s full name in the greeting, which is indicated by a coded field notation. The field notation autogenerated the patient’s full name from their chart when sent to the patient. The infographic and questionnaire are sent as attachments to the message. The self-report questionnaire results are returned (like a reply) to the Epic account that sent the initial message. The reply or results can be securely forwarded to other health care professionals with an Epic account, and the LCS scheduler can message the patient directly on MyChart to discuss eligibility and scheduling.

### Self-Referral Tool Pilot Test

#### Overview

A one-group pilot test was conducted to determine the uptake of LCS in response to the self-referral tool.

#### Study Setting and Participants

A convenience sample of medical record numbers (MRNs) was identified from another ongoing quality improvement study in the Medical University of South Carolina (MUSC) LCS program. The other study examined the uptake of LCS among women who had completed a mammogram in the past year. The data set included patients who were (1) network-affiliated, (2) female at birth, (3) between 50 and 77 years of age, (4) undergone a mammogram in the past year, (5) had no previous LCS care or history of lung cancer, and (6) had an active Epic MyChart account.

#### Study Procedures

MRNs for the pilot were extracted through random sampling. The MRNs listed on the data set were assigned a sequential number. The sequential number boundaries were set on a random sequence generator software, which produced a random order list. From the randomized list, the first 150 assigned numbers (and accompanying MRN) were selected to receive the self-referral tool.

The tool components (the message in Figure 1 linking to the questionnaire in Figure 2) were delivered to eligible patients through MyChart from a research-designated Epic account linked to a CRA. Once sent, patients would receive a notification that they had a new MyChart message and could access it through their secure Epic MyChart account; this service is accessible on a mobile app or through a desktop web browser (displayed in Multimedia Appendices 2 and 3).

#### Data Collection and Analysis

Responses to the self-referral questionnaire were collected and stored in the Epic account of the CRA, including tracking if the patient opened the message. The CRA tracked (1) the status of the message (open vs unopened); (2) the status of the questionnaire (completed vs not completed); (3) the completed questionnaire status (eligible vs ineligible); and (4) outcomes (LCS status and lung cancer diagnosis, if applicable). Percentages for categorical variables were analyzed.

### Ethical Considerations

The project was determined to be a quality improvement study by the Medical University of South Carolina Institutional Review Board for Human Research using their "IRB QI/Program Evaluation Self-Certification Tool" and therefore not subject to ethics board review or approval. Given the nature of the study, formal patient consent was also not obtained. Participants verbally consented to participate in the pretest portion of the study, during which the study tool was evaluated, and informal interviews were conducted. Participants did not receive financial remuneration for their participation. All data reported in this publication have been deidentified.

## Results

### Pretest Results

#### Participant Demographics

A total of 17 patients completed the survey for tool evaluation in October 2022. More than half were new patients (n=10, 60%), and the remaining were returning patients or annual screeners (n=7, 41%; Table 1). The majority were White (n=15, 88%), female (n=11, 66%), self-reported former smokers (n=11, 65%), and were referred to the LCS program from a network-affiliated primary care provider (n=14, 82%). The average age was 63.5 (SD 8.10), ranging from 52 to 77. Health insurance coverage among participants included Medicare (n=9, 53%) and private (n=8, 47%).

**Table 1:** Demographics and characteristics of pretest participants surveyed for self-referral tool evaluation (N=17).

Demographic		Value
**Age (years)**		
	Mean (SD)	63.5 (8.10)
	Minimum, maximum	52, 77
**Sex at birth, n (%)**		
	Female	11 (65)
	Male	6 (36)
**Race, n (%)**		
	Black or African American	2 (12)
	White	15 (88)
**Appointment type, n (%)**		
	New patient referral	10 (59)
	Returning (annual) screener	7 (41)
**Smoking status, n (%)**		
	Current smoker	6 (35)
	Former smoker	11 (65)
**Health insurance coverage, n (%)**		
	Medicare	9 (53)
	Private	8 (47)
**Referral pathway to LCS^a^ clinical care program, n (%)**		
	Network affiliated PCP^b^	14 (82)
	Network affiliated non-PCP	1 (6)
	Self-referral	2 (12)

^a^LCS: lung cancer screening.

^b^PCP: primary care provider.

#### Questionnaire

When surveyed about their understanding of the health topic in the message, 100% reported strongly agree or agree. When surveyed about their self-efficacy to complete the survey, 100% reported strongly agree or agree with the easiness of completion.

#### Engagement Message

When surveyed about their understanding of the health topic in the message, 100% reported strongly agree or agree. When surveyed about their understanding of the threat of lung cancer, 100% reported strongly agree or agree. When surveyed about their understanding of the benefit of LCS, 100% reported strongly agree or agree. Finally, when surveyed about their self-efficacy to take the LCS survey, 100% reported strongly agree or agree.

#### Infographic

When surveyed about their understanding of the health topic in the infographic, 100% reported strongly agree or agree. When surveyed about their perceived threat to lung cancer, 94% (n=16) reported strongly agree or agree, and 6% (n=1) reported neutral. When surveyed about their understanding of the benefit of LCS, 100% reported strongly agree or agree. Finally, we surveyed their self-efficacy to scan a QR code to access additional resources to learn more about lung cancer and LCS, 82% (n=14) reported strongly agree or agree, and 18% (n=3) reported strongly disagree.

#### Likelihood of Survey Completion

When asked if they would complete the self-referral survey sent via MyChart, the majority or 94% (n=16), responded yes and 6% (n=1) reported unsure. When asked if they would still fill it out if they did not receive the additional introductory message and infographic, the majority, 71% (n=12), reported that they would still complete the survey even without the additional materials, 41% (n=7) indicated they would prefer to receive the additional materials, and 29% (n=5) indicated they would complete any survey (ie, not health screening related) sent to them in MyChart. A total of 18% (n=3) indicated they were unsure, while the remaining 12% (n=2) indicated they would only complete the questionnaire if they received the additional materials.

#### Key Themes

A total of 15 participants provided oral feedback when gauged about their perceptions of the tool during the pretest survey. The most common themes that emerged from the data were poor self-efficacy in using the QR code, high self-efficacy in completing the survey, and heightened appreciation for information on LCS and the self-referral survey.

### Pilot Test Results

Of the 150 patients sampled, 48% (n=72) opened the message, and 28% (n=20) completed the survey. Based on the initial sample size, there was an overall response rate of 13% (n=20).

Of the 20 that completed the survey, 45% (n=9) self-reported LCS eligibility. Following referral to the LCS navigator, who completed an additional manual EHR review prior to scheduling, determined that 6 of the self-referring individuals were ineligible. The remaining 3 self-referring individuals were scheduled for LCS. At the time of analysis, 2 had attended an LCS-shared decision-making appointment and underwent a low-dose computer tomography scan (LDCT). The findings of the 2 completed LDCTs were categorized into lung computed tomography Screening Reporting and Data System (LUNG RADS) I and LUNG RADS III.

**Figure 3:**
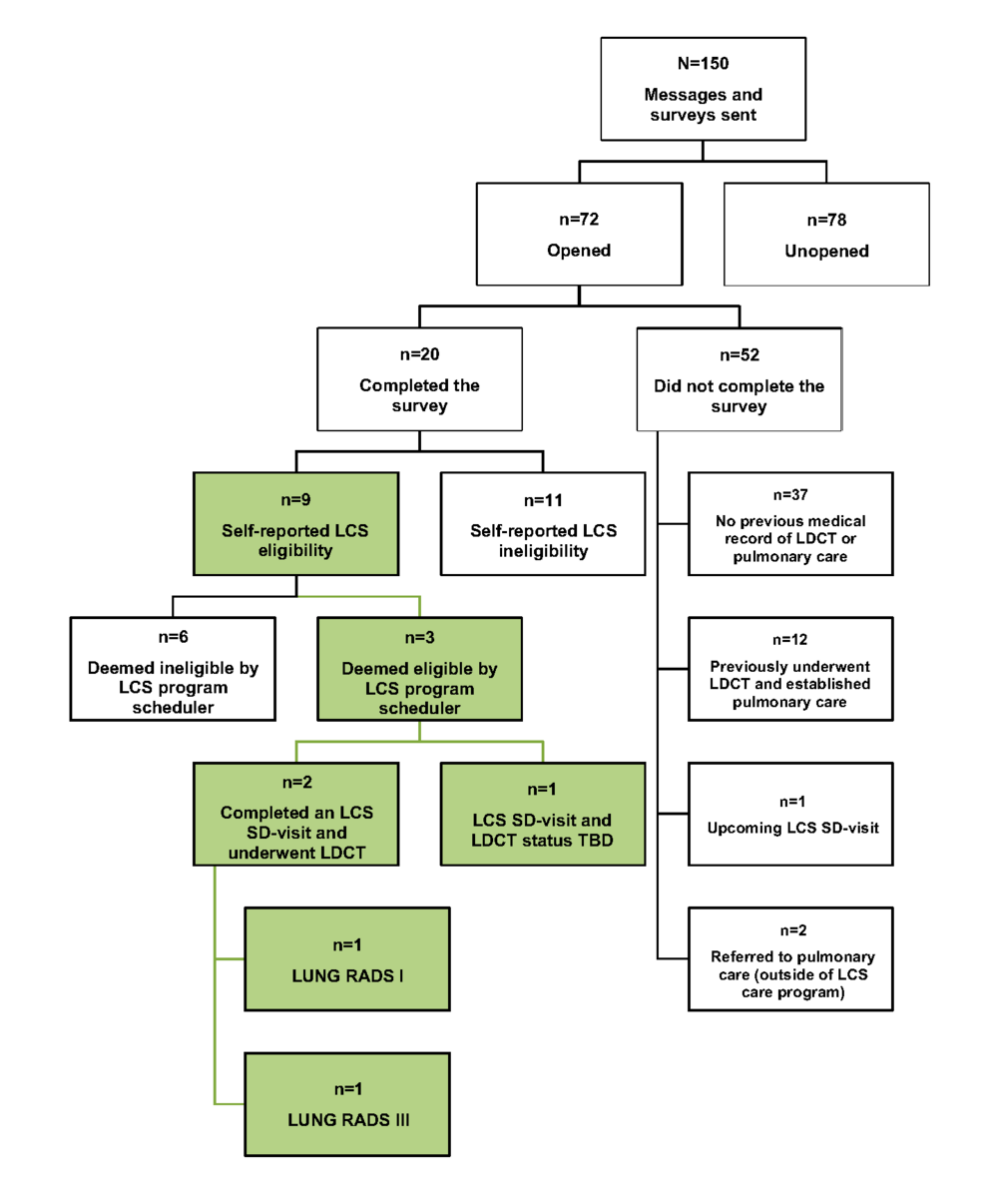
Initial application test consort diagram. LCS: lung cancer screening; LDCT: low-dose computer tomography scan; LUNG RADS: lung computed tomography screening reporting and data system; SD: shared-decision making; TBD: to be determined or LCS visit and results pending.

A manual EHR review was conducted of the 52 individuals who did not complete the survey after opening the message. Of those patients, 71% (n=37) had no previous record of LDCT or established pulmonary care, 23% (n=12) had previously undergone LDCT and established pulmonary care, 0.02% (n=1) had contacted the LCS program navigator to schedule screening after opening the message, and 0.04% (n=2) had self-referred to pulmonary care (outside of the LCS care program) after opening the message. A consort diagram of each patient’s phase in the tool application is displayed in Figure 3.

## Discussion

### Principal Findings

This formative process created an EHR self-referral tool for an established LCS program. The preimplementation evaluation of the tool indicates that the language, phrasing, and visualizations in the message, survey, and infographic were understood. Overall, a majority reported a favorable interpretation of the self-referral tool and related materials, indicating that this tool is a positive stimulus to trigger the decision-making process to accept a recommended health action (eg, getting an LCS). Indeed, results from the pilot test revealed that the tool successfully aided the establishment of pulmonary care for 3 patients with LCS.

While the materials were mostly favorably perceived by patients, there was minor confusion surrounding the QR code on the infographic. The data collected on the infographic suggested that patients perceived self-efficacy in using the QR code, but the qualitative data suggested poor self-efficacy. Further, there are logistical concerns about the QR code being viewed on a mobile phone which would result in people needing help to scan from the same device. The only modification to the pretested materials would involve removing the QR code from the infographic. A link could be provided in addition to the QR code which would allow the patient to directly navigate to the tool rather than using an additional device to do so.

Based on our experience with Epic software and the initial application results, this tool can successfully be integrated into an EHR and produce self-referrals to LCS. Although the overall response rate was low, nearly half (48%) of our patients viewed the initial engagement message, consistent with other results found in other EHR-based messaging cancer screening tools [17]. Many patients who received the tool and did not complete the questionnaire are still eligible for LCS, and more work is needed to improve engagement with the self-referral tool and subsequent LCS uptake. However, this low-cost automated tool has the potential to add to a more considerable effort to increase LCS uptake.

There are several limitations to the study that could limit generalizability. Interviews were not recorded and transcribed verbatim for accuracy but instead collected through notetaking, which impacts response accuracy due to implementation errors. Survey-related effects that may have limited conclusions were response bias, specifically social desirability bias and anchoring bias. The social desirability bias became evident when differences were noted between survey and interview responses regarding the QR code. Additionally, anchoring bias is apparent as new patients were surveyed after their shared decision-making appointment, and their answers could have been influenced by the knowledge of lung cancer and LCS obtained during that visit; moreover, this same bias is expected from returning patients surveyed. Returning patients were sampled in addition to new patients with LCS to increase feedback and compensate for the poor number of new LCS referrals. Further, the survey was administered by an interviewer face-to-face and not self-administered, so there is the potential for mode effect. Another limitation could lie in the characteristics of the pilot test sample. Participants for this study were selected through convenience sampling of another project that only included a sample of women who completed a mammogram. Expanding the scope of pilot-testing to engage racially, ethnically, and gender-diverse individuals who are referred to LCS from various clinical care teams or services (eg, primary care and gerontology) could identify optimal tool delivery approaches to increase engagement with and applicability of the tool.

### Conclusions

Pretest survey results indicate that the language, phrasing, and visualizations used in the tool are understood and increase the potential for engaging in LCS care. Most pretest participants reported favorably interpreting the self-referral tool and related components. This tool is a positive stimulus to trigger the decision-making process to accept a recommended health action (eg, getting an LCS). Nearly half (48%) of the initial application test population opened the message, with an overall response rate of 13%. This EHR-integrated tool offers an additional opportunity for referral to other clinical or ancillary services.

## Appendix

Multimedia Appendix 1 Lung cancer infographic mapped to HBM constructs.

Multimedia Appendix 2 MyChart mobile app and desktop webpage–patient point of view.

Multimedia Appendix 3 Self-referral pathway from LCS eligibility questionnaire.

## Abbreviations

CRA: clinical research assistant
EHR: electronic health record
HBM: Health Belief Model
LCS: lung cancer screening
LDCT: low-dose computer tomography
LUNG RADS: lung computed tomography screening reporting and data system
MRN: medical record number
MUSC: Medical University of South Carolina

## Data Availability

The data sets generated and analyzed during this study are available from the corresponding author on reasonable request.
