# Supplementary material for: Development of an Electronic Health Record Self-Referral Tool for Lung Cancer Screening: One-Group Posttest Study
Source: JMIR Form Res. 2024 Jun 12;8:e53159. doi: 10.2196/53159 (PMC11208829; doi:10.2196/53159)
Supplement: Multimedia Appendix 1 [file formative_v8i1e53159_app1.pdf]

[HEALTHCARE SYSTEM/HOSPITAL LOGO]

LUNG CANCER SCREENING

Perceived Severity

Lung cancer is the #1 cancer killer.

Lung cancer kills more people in the United States than breast, colon, and prostate cancer combined.

Without screening, 70% of lung cancers are found at a later stage when there is little chance for a cure.

This is true for both men and women.

1 in 16 people in the US will be diagnosed with lung cancer in their lifetime.

Perceived Benefit

Early detection through a low-dose CT is essential to beating lung cancer.

Perceived Susceptibility

Am I at Risk?

Are you between the ages of 50 and 77?

Do you currently smoke or have quit within the past 15 years?

Is your 'pack year' number greater than 20?

If you answered "Yes" to each of these questions you may be at high risk for developing lung cancer.

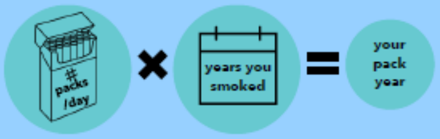

Not everyone who meets the initial criteria will be eligible for screening. Our team will help guide you as to whether screening can benefit you or not.

At least 14 million Americans qualify as at risk for lung cancer and are recommended by the USPSTF to receive annual screening with low-dose CT.

Cue to Action

If you think you or someone you know is at risk for lung cancer and would like to know more about screening eligibility, please scan the QR code or call (###)-###-#### to learn more about the [CLINIC/DEPARTMENT/ PROGRAM].

QR CODE LINKED TO LCS SERVICES OFFERED AT [HEALTHCARE SYSTEM/HOSPITAL]
