# Supplementary material for: Development of an Electronic Health Record Self-Referral Tool for Lung Cancer Screening: One-Group Posttest Study
Source: JMIR Form Res. 2024 Jun 12;8:e53159. doi: 10.2196/53159 (PMC11208829; doi:10.2196/53159)
Supplement: Multimedia Appendix 2 [file formative_v8i1e53159_app2.docx]

**Multimedia Appendix 2.** MyChart mobile app and desktop webpage–patient point of view.
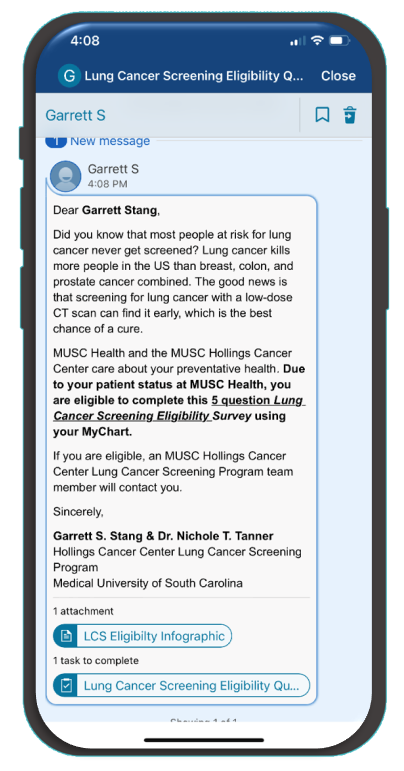


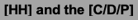

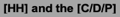

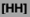

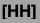

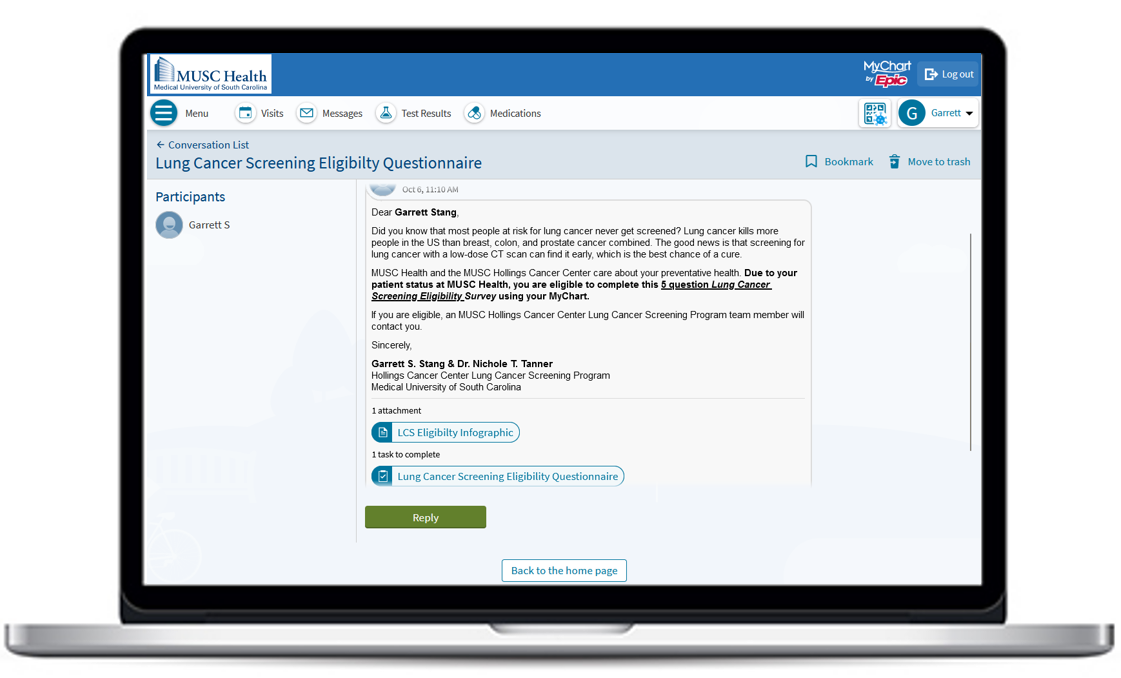


**John**

**[HH]**

**[HH]**

**[HH] LOGO**

**[HH]**

**John Smith,**

**John Smith,**

**John S**

**John S**

**[CLINICAL TEAM MEMBER(S)]**

[CLINIC/DEPARTMENT/PROGRAM]

[HEALTHCARE SYSTEM/HOSPITAL]

**[CLINICAL TEAM MEMBER(S)]**

[CLINIC/DEPARTMENT/PROGRAM]

[HEALTHCARE SYSTEM/HOSPITAL]

**[C/D/P]**

**[HH] and the [C/D/P]**

**John S**
