# Supplementary figures and images for: Development of an Electronic Health Record Self-Referral Tool for Lung Cancer Screening: One-Group Posttest Study
Source: JMIR Form Res. 2024 Jun 12;8:e53159. doi: 10.2196/53159 (PMC11208829; doi:10.2196/53159)

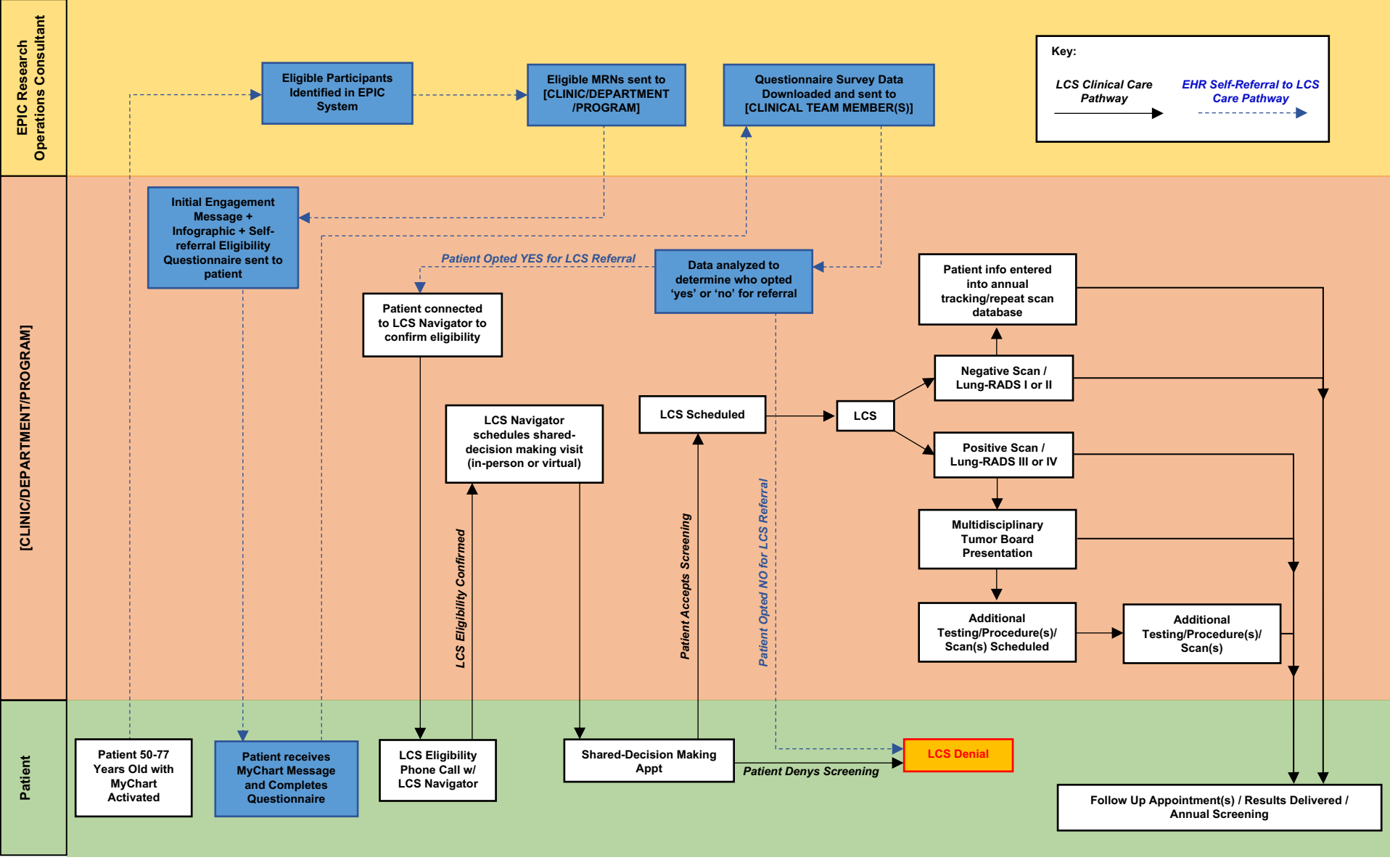

Supplement: Multimedia Appendix 3 [file formative_v8i1e53159_app3.pdf]
